# Lower values of a novel index of Vagal-Neuroimmunomodulation are associated to higher all-cause mortality in two large general population samples with 18 year follow up

**DOI:** 10.1038/s41598-021-82168-6

**Published:** 2021-01-28

**Authors:** Marc N. Jarczok, Julian Koenig, Julian F. Thayer

**Affiliations:** 1 Department of Psychosomatic Medicine and Psychotherapy, Ulm University Medical Center, Albert-Einstein-Allee 23, 89070 Ulm, Germany; 2 Section for Experimental Child and Adolescent Psychiatry, Department of Child and Adolescent Psychiatry, Centre for Psychosocial Medicine, Heidelberg University, Heidelberg, Germany; 3 University Hospital of Child and Adolescent Psychiatry and Psychotherapy, University of Bern, Bern, Switzerland; 4 Department of Psychological Science, The University of California, Irvine, CA USA

**Keywords:** Predictive markers, Neurophysiology, Neuroimmunology, Peripheral nervous system

## Abstract

In recent clinical practice, a biomarker of vagal neuroimmunomodulation (NIM), namely the ratio of vagally-mediated heart rate variability (vmHRV) and CRP, was proposed to index the functionality of the cholinergic anti-inflammatory pathway. This study aims to transfer and extend the previous findings to two general population-based samples to explore the hypothesis that NIM-ratio is associated with all-cause mortality. Two large population studies (MIDUS 2: N = 1255 and Whitehall II wave 5: N = 7870) with complete data from a total of N = 3860 participants (36.1% females; average age = 56.3 years; 11.1% deaths, last exit 18.1 years post inclusion) were available. NIM indices were calculated using the vagally-mediated HRV measure RMSSD divided by measures of CRP (NIM_CRP_) or IL-6 (NIM_IL6_). The NIM-ratios were quartiled and entered into age, ethnicity and body mass index adjusted Cox proportional hazards models. For NIM_IL6_ the lowest quartile was 45% more likely to die during the observed period (max. 18 years follow-up) compared to the highest quartile (HR = 0.55 CI 0.41–0.73; *p* < .0001). NIM_CRP_ parallel these results. Here we show that an easily computable index of IL-6 inhibition is associated with all-cause mortality in two large general population samples. These results suggest that this index might be useful for risk stratification and warrant further examination.

## Introduction

Elevated pro-inflammatory cytokines such as interleukin-6 (IL-6) and C-reactive protein (CRP) have been seen in patients with Coronavirus Disease 2019 (COVID-19) and are associated with worse outcomes^1^. Furthermore, a systematic review and meta-analysis demonstrated serum levels of IL-6 to be significantly elevated in the setting of complicated COVID-19 disease, and increased IL-6 levels to be in turn significantly associated with adverse clinical outcomes^2^. Consequently, the Society for Immunotherapy of Cancer has suggested that anti-IL-6 drugs may be useful in COVID-19 treatment^3^. Beside this acute drug treatment option, the human body has several anti-inflammatory mechanisms including the cholinergic anti-inflammatory reflex involving efferent vagus nerve activity to modulate inflammatory responses. The pivotal role of efferent vagal activity, as indexed by vagally-mediated heart rate variability (vmHRV), has been described and termed the cholinergic anti-inflammatory pathway (CAP)^4^. The CAP modulates inflammation, e.g., by inhibiting the HMGB1 and cytokine release, via the macrophages’ α7 nicotinic acetylcholine receptor (α7nAChR)^4,5^. This CAP has been identified as highly relevant in COVID-19-patients hypothetically with detailed mechanism^6,7^, in clinical practice^8^, and it´s modulation through indirect methods such as physical activity and neuromodulation^9,10^. However, the CAP has played an important role in the pre-covid era and outside an acute clinical setting. A recent meta-analysis clearly demonstrated that vmHRV is inversely associated in a relevant manner with both circulating levels of IL-6 and CRP in short- and long-term studies^11^. In addition, we demonstrated that the level of vmHRV at baseline predicts CRP levels in at 4 year follow up in healthy adults^12^.

In recent clinical practice, a biomarker of vagal neuroimmunomodulation (NIM), namely the ratio of vmHRV and CRP, was evaluated in two samples of patients with fatal cancer^13^. A greater NIM ratio at baseline was prospectively related not only to a reduced tumor growth rate but also to a longer survival in both samples^13^. The authors of the cancer studies hypothesize that this NIM-ratio may index proper function of the CAP in cancer populations. The primary research question of this investigation is as follows: Is the NIM ratio also an indicator of mortality in the general population? Given that IL-6 is a more proximate measure on the CAP compared to CRP, the second aim is to compare the NIM_CRP_ ratio with the NIM_IL6_ ratio.

By illustrating the general feasibility and clinical utility of such an index, we argue that it might have important clinical implications in the assessment and risk stratification of employees such as hospital personnel and the general population at risk for cytokine release syndrome (CRS) as seen in SARS-CoV-2 infections. We have recently shown that vmHRV may be a useful tool for risk stratification including elevated levels of CRP^14^. This might also be true for the NIM-ratio.

## Results

A total of N = 3860 participants from MIDUS 2 (23.9%) and Whitehall II (76.1%) with complete data were included (see Table 1). This analysis sample comprised of 36.1% females (MIDUS 2 = 55.1%; Whitehall = 30.1%), 7.9% none-white participants (MIDUS 2 = 5.8%; Whitehall = 8.5%) with an average age of 56.3 years. In sum, a total of 429 deaths (11.1%) from all-cause mortality were recorded (MIDUS 2 = 8.8%; Whitehall = 11.8%). The last exit was observed 18.1 years post inclusion. Both NIM-values (NIM_CRP_ & NIM_IL-6_) of each quartile were of equivalent magnitude in the datasets (Table 1). Heterogeneity measures indicated no heterogeneity for the NIM_CRP_ model (I^2^ = 0.0%, Cochran's Q = 0.68; df1, *p* = 0.41) and NIM_IL6_ model (I^2^ = 0.0%, Cochran's Q = 0.44; df1, *p* = 0.51).

**Table 1:** Descriptive statistics by datasets.

	MIDUS 2 (N = 922)		Whitehall T5 (N = 2938)		Total (N = 3860)	
	Mean	SD	Mean	SD	Mean	SD
**Age (Years at baseline)**	57.5	11.3	55.9	6.09	56.3	7.69
**BMI (kg/m**^**2**^**)**	29	5.8	26.2	3.99	26.9	4.64
**CRP (ug/ml)**	2.55	3.74	2.18	3.56	2.27	3.6
**IL-6 (pg/ml)**	2.67	2.59	1.9	1.54	2.08	1.88
**Survival (Month)**	147	25.2	201	31.6	188	37.8
**RMSSD (ms)**	10.7	8.43	24.8	22.6	21.4	21
**Female**	55.1%		30.1%		36.1%	
**Ethnicity non-white**	5.8%		8.5%		7.9%	
**Deaths**	8.8%		11.8%		11.1%	
**NIM-IL6**						
**1. Quartile**	**(N = 230)**		**(N = 741)**		**(N = 971)**	
**NIM-IL6**	.902	.0565	.925	.053	.919	.0547
**RMSSD (msec)**	5.1	2.77	14.3	8.46	12.2	8.48
**IL-6 (pg/ml)**	4.86	4.07	3.55	2.18	3.86	2.8
**2. Quartile**	**(N = 235)**		**(N = 742)**		**(N = 977)**	
**NIM-IL6**	.981	.0125	.987	.00937	.986	.0105
**RMSSD (msec)**	7.74	3.75	16.4	7.24	14.3	7.54
**IL-6 (pg/ml)**	2.47	1.38	1.64	.564	1.84	.909
**3. Quartile**	**(N = 236)**		**(N = 723)**		**(N = 959)**	
**NIM-IL6**	1.02	.012	1.02	.00879	1.02	.00996
**RMSSD (ms)**	10.3	2.67	22.8	7.41	19.7	8.5
**IL-6 (pg/ml)**	1.81	.917	1.27	.503	1.4	.672
**4. Quartile**	**(N = 221)**		**(N = 732)**		**(N = 953)**	
**NIM-IL6**	1.08	.0322	1.08	.0867	1.08	.0775
**RMSSD (msec)**	20	11.8	45.8	35.2	39.8	33.2
**IL-6 (pg/ml)**	1.51	.825	1.12	.586	1.21	.669
**NIM-CRP**						
**1. Quartile**	**(N = 238)**		**(N = 745)**		**(N = 983)**	
**NIM-CRP**	.911	.046	.934	.0526	.929	.0521
**RMSSD (msec)**	5.17	3.02	13.6	8.47	11.6	8.35
**CRP (ug/ml)**	5.42	5.99	5.42	5.66	5.42	5.74
**2. Quartile**	**(N = 235)**		**(N = 741)**		**(N = 976)**	
**NIM-CRP**	.98	.0121	.989	.0066	.987	.00912
**RMSSD (msec)**	7.31	2.39	15.9	6.64	13.8	6.95
**CRP (ug/ml)**	2.04	1.91	1.34	1.26	1.51	1.47
**3. Quartile**	**(N = 232)**		**(N = 718)**		**(N = 950)**	
**NIM-CRP**	1.02	.0111	1.01	.0078	1.01	.00928
**RMSSD (msec)**	10.6	3.01	23.1	5.97	20	7.6
**CRP (ug/ml)**	1.44	1.39	.975	.953	1.09	1.09
**4. Quartile**	**(N = 217)**		**(N = 734)**		**(N = 951)**	
**NIM (RMSSD/CRP)**	1.07	.0317	1.07	.0859	1.07	.0769
**RMSSD (msec)**	20.4	11.7	46.7	34.7	40.7	32.9
**CRP (ug/ml)**	1.14	1.2	.913	1.18	.964	1.18

Log-rank tests testing for equality of survivor functions were all *p* < 0.001 (see Table 2).

**Table 2:** Cox proportional hazards models (Observations N = 3860, No. of deaths = 429).

NIM (RMSSD/IL-6)	Haz. Ratio	Std. Err	z	*P* >|z|		[95% Conf. Interval]
**1. Quartile (low)**	Ref					
**2. Quartile**	.619	.075	− 3.959	< 0.001	.488	.785
**3. Quartile**	.427	.065	− 5.629	< 0.001	.318	.574
**4. Quartile (high)**	.547	.080	− 4.137	< 0.001	.410	.728
**Age (Years; centered)**	1.107	.008	13.755	< 0.001	1.091	1.123
**BMI (kg/m**^**2**^**; centered)**	.883	.161	− .679	.497	.617	1.264
**Ethnicity non**-**white**	1.021	.012	1.810	.070	.998	1.045
*Log rank test*	*chi*^2^* (3)* =	*108.64*	*p* > *chi*^2^	< *0.001*		

NIM (RMSSD/CRP)	Haz. Ratio	Std. Err	z	P > |z|	[95% Conf. Interval]	
**1. Quartile (low)**	Ref					
**2. Quartile**	.603	.076	− 4.006	< 0.001	.471	.772
**3. Quartile**	.583	.081	− 3.872	< 0.001	.443	.766
**4. Quartile (high)**	.566	.081	− 3.986	< 0.001	.428	.749
**Age (Years; centered)**	1.111	.008	14.452	< 0.001	1.095	1.127
**BMI (kg/m**^**2**^**; centered)**	1.025	.012	2.072	.038	1.001	1.049
**Ethnicity non**-**white**	.931	.170	− .391	.696	.651	1.332
*Log rank test*	*chi*^2^*(3)* =	*61.46*	*p* > *chi*^2^	< *0.001*		

Models were stratified by dataset (Whitehall II; MIDUS 2) and sex (male; female) NIM: Neuroimmunomodulatory index (RMSSD/CRP or RMSSD/IL-6). Haz.Ratio = Hazard Ratio; Std. Err. = Standard Error.

**Reading example**: A white participant with an average age and BMI in the 4th Quartile group of NIM-IL6 were 45.3% less likely to die compared to participants from the 1st NIM-IL6 Quartile group, with a 95% confident between 27.2 and 59%. (i.e. we are 95% sure that participants in the Q4 group were between 27.2% and 59% less likely to die than participants in the Q1 group).

**Figure 1:**
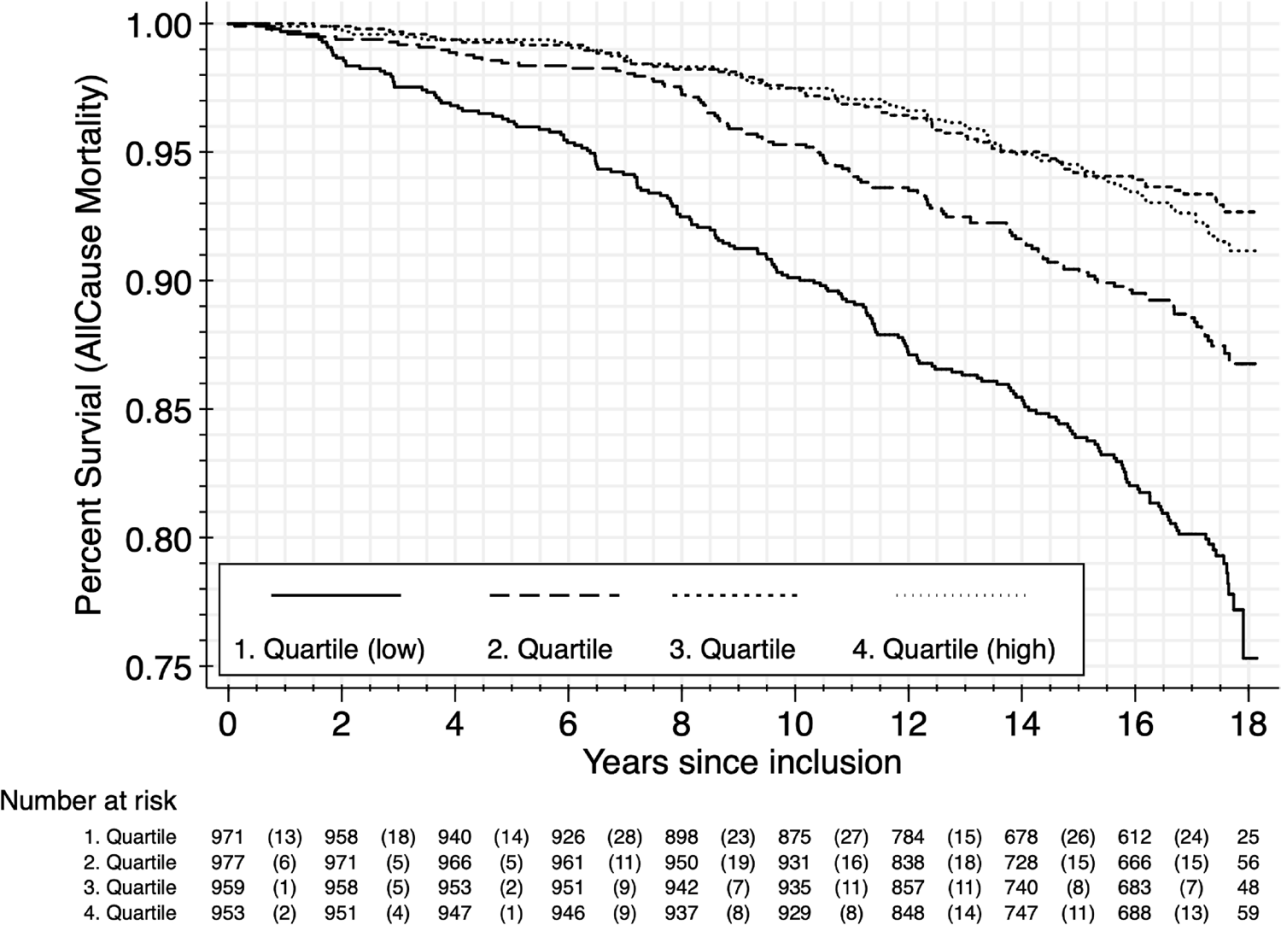
Kaplan–Meier survival function by NIM_IL6_ quartile (Observations N = 3860, No. of deaths = 429). At the End of the observation period (18 years) 75% survived in the belonging to the lowest quartile of NIM-Ratio (0.666–0.969) and 91% survived in the highest quartile of NIM-Ratio (1.033–1.996) (Log-rank test for equality of survivor functions: *chi*^2^ (3) 108.64; *p* < 0.001).

**Figure 2:**
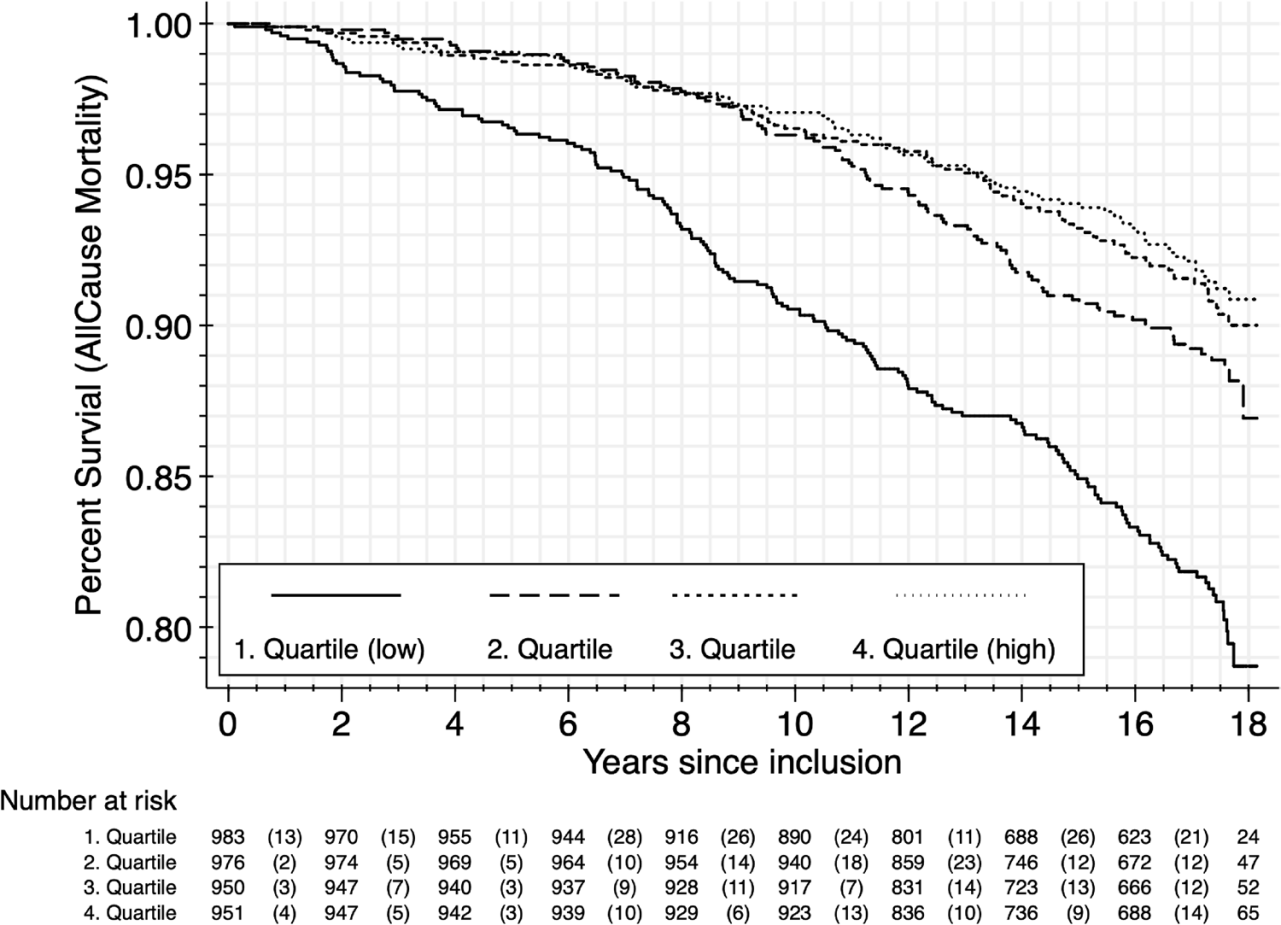
Kaplan–Meier survival function by NIM_CRP_ quartile (Observations N = 3860, No. of deaths = 429). At the End of the observation period (18 years) 79% survived in the belonging to the lowest quartile of NIM-Ratio (0.594–0.977) and 91% survived in the highest quartile of NIM-Ratio (1.027–2.001) (Log-rank test for equality of survivor functions: *chi*^2^(3) 68.51; *p* < 0.001).

Both NIM_IL6_ and NIM_CRP_ ratios showed the hypothesized negative association with survival time in the multiple adjusted Cox regression models (see Table 2). Here, the lowest quartile in the NIM_IL6_ model was 45% more likely to die during the observed study period compared to the highest quartile (Hazard Ratio [HR] = 0.55; 95% Confidence Interval [CI] 0.41–0.73; *p* < 0.0001; see Fig. 1). Results were nearly identical for NIM_CRP_ (HR = 0.57; CI 0.43–0.75; *p* < 0.0001; see Fig. 2). The AIC and BIC of the NIM_IL-6_ regression model was: 5721.368 and 5758.918, respectively. Similarly, AIC and BIC of the NIM_CRP_ regression model was 5735.883 and 5773.433, respectively.

Ethnicity (white vs. non-white) had no statistically relevant impact on survival in both models (see limitation section). With increasing age (per year), the average death risk increases by 10.7% (95% CI 9.1–12.3% or HR = 0.1.107; CI 1.091–1.123; *p* < 0.0001) for white participants with an average BMI in the lowest quartile group (1st) in the NIM_IL6_ model. For the same group NIM_CRP_ model the per year death risk increases by 11.1% (95% CI 9.5–12.7% *p* < 0.0001)). Similarly, every BMI unit increase (kg/m^2^) is associated with a 2.5% increased risk of death (95% CI 0.1–4.9%; *p* = 0.038) NIM_CRP_ model but not in the NIM_IL6_ (Table 2).

## Discussion

Results showed a negative association between NIM and mortality – the smaller the NIM ratio the shorter the survival time in two large general population samples. Hence, the NIM ratio is an indicator of mortality not only in the cancer samples as demonstrated by^13^, but also in the present two general population samples. Comparing the Cox regression model fit of the NIM_CRP_ ratio with the NIM_IL6_ ratio revealed a slightly better performance of the NIM_IL6_ Cox-regression. In accordance with previous literature, age in both and BMI in CRP model were associated with an increased risk of death. This effect was statistically independent of the risk quartile group. Interestingly, BMI was not a significant contributor to death risk in the NIM_IL6_ model. Yet, IL-6 is elevated^15^ and RMSSD is decreased^16^ with increasing BMI levels. Therefore, it might be assumed that BMI differences are statistically enclosed in the NIM_IL6_ quartiles (i.e. shared variance), but detailed analysis are beyond the scope of this report.

The autonomic nervous system (ANS) is known to be involved in the regulation of innate immune responses and inflammation via the CAP^4,5^. Specifically, the efferent vagally mediated pathway regulates inflammation and pro-inflammatory cytokine release such as IL-6 from acetylcholine-synthesizing T-cells^4,5,17^. Accordingly, plasma levels of pro-inflammatory cytokines increase in cervical or subdiaphragmatic vagotomy, while vagal stimulation or administration of acetylcholine decrease IL-6 cytokine levels^5,17–19^. In this bidirectional brain-body communication, the α7nAChR is an essential receptor^5,18^. Consequently, several α7nAChR agonists have been identified as experimental anti-inflammatory therapeutics with potential for clinical development^18^. Here, cholinergic agonists can inhibit cytokine synthesis and protect against cytokine-mediated diseases such as the cytokine release syndrome (CRS, also described as cytokine storm) seen in COVID-19 patients^20^. Importantly, the cytokine profile described in COVID-19-Patients shows large similarities with the cytokine profile of α7nAChRs dysregulated macrophages i.e. massively secreting IL-1β, IL-6, tumor necrosis factor alpha (TNF α) α and IL-18 among others^20–22^. In consequence, the stimulation of the vagus nerve may prevent the damaging effects of cytokine release in experimental sepsis, endotoxemia, ischemia/reperfusion injury, hemorrhagic shock, arthritis, and other inflammatory syndromes. Several medical hypothesis papers address potential clinical usage of the CAP, particularly Leitzke et al. detail the underlying mechanisms in the SARS-CoV-2 infections^6^. Therefore, assessing the NIM in COVID-19-patients at hospitalization might be an additional, useful risk indicator to identify patients at high risk for worsening COVID-19-symptoms such as CRS or co-infections.

The present study and the previous study in two acute cancer populations^13^ demonstrate that the NIM-ratio works as risk stratification tool on different time scales (several weeks vs. 18 years) and different populations (general vs. cancer). Therefore, the NIM-ratio might also be relevant for risk stratification in patients at elevated risk for CRS such as hospitalized COVID-19 patients within a 2–3-week timeframe, in which more than 75% of patients are released from hospital^1^.

IL-6 (compared to CRP) represents a downstream but more proximate measure of this reflex arc and might be more suitable for NIM calculations in general population settings and therefore more sensitive. Importantly, this neural reflex has much shorter response times compared to humoral anti-inflammatory pathways such as cortisol release via the hypothalamic–pituitary–adrenal axis^5^ and thus may have a high clinical relevance with respect to the etiology of inflammation related CRS-morbidity and mortality. For example, the noninvasive, transcutaneous stimulation of the auricular branch of the vagus nerve (tragus stimulation located at the external ear) has been shown to decrease circulating markers of inflammation such as TNF-α and CRP^23,24^ and might be a promising target. A rat model of baroreceptor denervation reduces the inflammatory burden (measurements of inflammatory cytokines such as IL-6; IL-10 and TNF-α in plasma and spleen) but worsened hemodynamic collapse^25^.

There are some limitations and risk of biases to this study. While the samples comprised of large cohort studies with long follow up times, it cannot be ruled out that participants with worse health at baseline were more likely to not participate at all or at the specific health monitoring (such as blood draw and EGC), although both cohorts recruited broadly. Similar, the other ethnicity group labeled “non-white” compared to “European or white” has its limitation because it represents a very heterogeneous group. Yet, due to limitations in data collection, no further information is available and/ or subgroups are too small for proper analysis.

While the present analysis investigates NIM at baseline with death for years of follow up, and the manuscript by Gidron et al. show associations on a time scale of months, the predictive power of day-to-day change e.g. in CRS is unknown.

## Conclusion

Here we show that an easily computable index of vagal IL-6 inhibition is associated with all-cause mortality in two large general population samples. These results suggest that this index might be useful indicator for risk stratification of both patients and general population.

## Methods

### Study populations

Two large population studies (MIDUS 2: N = 1255 & Whitehall II wave 5: N = 7870) were available for analysis. The primary aim is to transfer the clinically applied NIM to general population samples. Both studies recruited broadly, have an investigative study team to determine alive status and protocols are available online. The authors confirm that all methods were performed in accordance with the relevant guidelines and regulations. The individual studies gained local ethical approval at their according institutions.

#### MIDUS 2

Open access data from the biomarker project of the second wave (2004–2009) of the *“Midlife Development in the U.S.”* study (MIDUS 2, P4; N = 1255) were matched with survival information from the MIDUS core mortality dataset until June 2019 (median follow-up = 12.25 years). According to the study description, institutional review board approval was obtained prior to study start and informed consent was obtained from each participant prior to enrolment in the study. Additional study details and data are publicly available from the official Website after registration (https://midus.colectica.org). Two supine 5-min electrocardiograms (ECG) were obtained during rest and vmHRV measures were derived per 5-min interval and averaged for this analysis. In cases were just one 5-min interval was valid or available, the single 5-min interval was used.

#### Whitehall II

Data from the fifth (1997–1999) phases of the UK Whitehall II longitudinal population-based cohort study were analyzed with a median follow-up of 17.2 years (N = 7870 participants in wave 5) and end of follow up June 2015. This ongoing cohort study of subjects initially targeted London-based British civil service office staff, aged 35–55 years^26^. The ECG recordings were made at the fifth (1997–1999) wave. A 5-min supine resting 12-lead ECG (KardiosisTM ECG acquisition module, Tepa, Inc., Turkey and Getemed ECG recorder, Getemed Teltow, Germany) was obtained after 5-min of rest and vmHRV measures were calculated. The University College London ethics committee approved the study and participants gave informed consent. Whitehall II data, protocols and other meta-data are available to bona fide researchers for research purposes (data sharing policy is available at https://www.ucl.ac.uk/epidemiology-health-care/data-sharing-faq). The present study included participants with data on vmHRV, inflammatory markers (CRP and IL-6), age, sex, ethnicity, and body mass index at phase 5 (1997–1999), as well as survival information from phases 11 (2012–2013, last updated in June 2015).

### Measurement and statistical analysis

The NIM indices were calculated as the ratio of the z-transformed autonomic parameter namely the root mean squared successive difference of interbeat intervals (RMSSD) to one of the z-transformed inflammatory markers (CRP or IL-6) stratified by dataset. This ratio is entered as quartiles into Cox-regression models to determine differences in survival time between groups. Survival was defined as time from baseline (month and year of enrollment) till death from all-cause mortality or till the end of follow-up. Calculations were stratified by sex (male; female) and dataset (MIDUS and Whitehall) and summarized using a two-stage individual participant-data (IPD) approach (i. e. meta-analysis). The Cox-regression models are additionally adjusted for age (years), ethnicity (white vs. non-white), and body mass index (kg/m^2^). The statistical criterion for model selection of Cox regressions were Akaike information criterion and Bayesian information criterion (BIC).

Data management and analyses were conducted using Stata (v15.1 SE, College Station, TX: StataCorp LP).

### Ethics approval and consent to participate

This publication relies on open access data. Each study obtained ethical approval and inform consent prior to data collection (please see method section).

### Consent for publication

Not applicable.

## Data Availability

The datasets supporting the conclusions of this article are available as follows: *“Midlife Development in the U.S.”* MIDUS 2 Biomarker Project (P4) and information from the MIDUS core mortality dataset until June 2019 are available from the MIDUS Colectia Portal under https://midus.colectica.org. 2019. This portal does not provide a unique identifier or permanent link or versioning of the data. Only the latest data are available. Additional Data and documentation are available from the Inter-university Consortium for Political and Social Research (ICPSR) under https://www.icpsr.umich.edu. However, the latest versions of the datasets are published at Colectia, and with some delay at ICPSR. Data was retrieved on February 20th 2020. Whitehall II data, protocols and other meta-data are available to bona fide researchers for research purposes (data sharing policy is available at https://www.ucl.ac.uk/epidemiology-health-care/data-sharing-faq). The present study included participants from phase 5 (1997–1999), as well as survival information from phases 11 (2012–2013, last updated in June 2015).

## Abbreviations

AIC: Akaike information criterion
ANS: Autonomic nervous system
BIC: Bayesian information criterion
BMI: Body mass index (kg/m^2^).
CAP: Cholinergic anti-inflammatory pathway
CI: Confidence Interval
CRP: C-reactive protein
CRS: Cytokine release syndrome
ECG: Electrocardiograms
HR: Hazard ratio
IL-6: Interleukin-6
IPD: Individual participant-data
MIDUS: Midlife Development in the U.S.
NIM: Neuroimmunomodulation
RMSSD: Root mean squared successive difference of interbeat intervals
TNF α: Tumor necrosis factor alpha
vmHRV: Vagally-mediated heart rate variability
α7nAChR: α7 Nicotinic acetylcholine receptor
